# An autoimmune disease prevented by anti-retroviral drugs

**DOI:** 10.1186/1742-4690-8-91

**Published:** 2011-11-08

**Authors:** Gabriele B Beck-Engeser, Dan Eilat, Matthias Wabl

**Affiliations:** 1 Department of Microbiology and Immunology, University of California, San Francisco, CA 94143-0414, USA; 2 Department of Medicine, Hadassah University Hospital and The Hebrew University Faculty of Medicine, Jerusalem 91120, Israel

**Keywords:** Aicardi-Goutières syndrome, myocarditis, Trex1, reverse transcriptase inhibitors

## Abstract

**Background:**

Both Aicardi-Goutières syndrome, a Mendelian mimic of congenital infection, and the autoimmune disease systemic lupus erythematosus can result from mutations in the gene encoding the enzyme Trex1. In mice, the absence of Trex1 causes severe myocarditis. The enzyme is thought to degrade endogenous retroelements, thus linking them to autoimmune disease. However, inhibition of reverse transcription by the inhibitor zidovudine (AZT) did not ameliorate the disease, weakening the link to retroelements.

**Findings:**

Here, we show that two other FDA-approved drugs that inhibit reverse transcriptase can ameliorate the myocarditis in Trex1-null mouse.

**Conclusions:**

The result suggests that retroelements contribute to this hereditary form of autoimmunity, and that treatment with retroelement inhibitors might ameliorate Aicardi-Goutières syndrome in humans.

## Findings

Aicardi-Goutières syndrome (AGS) [1] is a genetically-determined encephalopathy with remarkable phenotypic overlap with the sequelae of congenital infection. Systemic lupus erythematosus (SLE) is an autoimmune disease characterized by the production of autoantibodies that target nucleic acids and their associated proteins. Like AGS [2], SLE is associated with a perturbation of type I interferon metabolism [3]. Both AGS [4], and a cutaneous subtype of SLE called familial chilblain lupus [5,6], can result from mutations in *TREX1*. Furthermore, mutations in *TREX1 *represent the single most common cause of monogenic SLE identified to date [7].

Trex1 is a ubiquitous DNA 3' exonuclease [8] that can degrade retroelements (retroviruses and retrotransposons) [9-11]. In Trex1-deficient mice, single-stranded DNA [12] derived from retroelement cDNA [9] accumulates in the cytoplasm of cells in the heart and is thought to trigger the sterile inflammatory myocarditis [13]. On the basis that unrestricted retroelements may cause, or at least contribute to, the disease [9], it was reasoned that it ought to be possible to treat or prevent disease with anti-retroviral agents. However, treatment of the mice with the reverse transcription inhibitor azidothymidine (AZT) did not rescue the mice from lethality [9]. It was argued that the absence of Trex1 may unleash hundreds of diverse reverse transcriptases encoded by the mouse genome, some of them being AZT resistant [9]. As a single agent, AZT also may leave some retroelements out of its range of activity. Finally, although it leads to premature termination of cDNA synthesis, AZT has only little effect on the synthesis of short reverse transcription intermediates, including those of spliced retroelement products [14,15]. The interrupted or slowed reverse transcription may create persistent exposure to cytoplasmic DNA products that elicit an antiviral innate immune response [16] coordinated by activation of type I IFNs (the so-called IFN-stimulatory DNA response [17]). Along this line, raltegravir, a drug that inhibits retroviral integrase and thus increases the concentration of cDNA in the cell, also exacerbates autoimmune disease [10].

**Figure 1:**
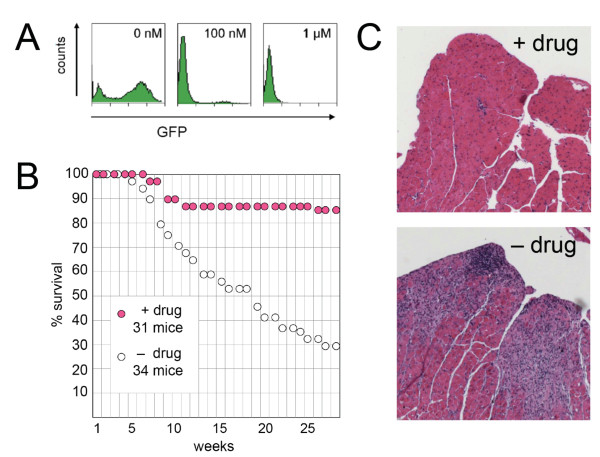
**Effect of reverse transcriptase inhibitors on survival of Trex1-deficient mice**. A) Inhibition of MLV cDNA synthesis by Truvada/Viramune. Flow cytometry graphs displaying GFP intensity generated by provirus: y-axis, cell number; x-axis, fluorescence intensity on a logarithmic scale. An MLV-based vector encoding GFP was added to NIH/3T3 cell cultures with 0, 100 nM, or 1 μM. B) Survival curves showing the effect of Truvada/Viramune (+ drug; magenta circles) on Trex1-deficient mice [13] obtained from D. Stetson [9]. The drugs were given from conception via the drinking water as a solution of 3 × 10^-4 ^M nevirapine, 1.6 × 10^-4 ^M emtricitabine and 9.4 × 10^-5 ^M tenofovir. Log rank test for the drug effect, p = 0.000014. C) Hematoxylin-eosin stained sections of the left heart ventricle of treated (+ drug) and non-treated (- drug) mice killed at 9 and 7 months of age, respectively. Sections from three mice were examined in each category.

In Trex1 deficient mice, the inflammation of the heart muscle takes an aggressive course, with mice starting to die after 4 weeks of age **(**Figure 1**)**. We sought to prevent the autoimmune disease with anti-retroviral drugs other than AZT. Keeping in mind that a single drug may leave some retroelements out of its range of activity, we decided to use a combination of drugs that inhibit reverse transcriptase. Because nucleoside reverse transcription inhibitors also inhibit human LINE-1 retrotransposition [18], we assumed that a Truvada/Viramune combination (both FDA-approved drugs) would inhibit both classes of retroelements--retroviruses and retrotransposons. Truvada is a fixed-dose combination tablet containing emtricitabine and tenofovir disoproxil fumarate [19]. Emtricitabine is a synthetic nucleoside analog of cytidine. Tenofovir disoproxil fumarate is converted in vivo to tenofovir, an acyclic nucleoside phosphonate (nucleotide) analog of adenosine 5'-monophosphate. Viramune (nevirapine) [20] blocks the reproduction of retrovirus earlier in its cycle than Truvada. It binds directly to reverse transcriptase and blocks the RNA-dependent and DNA-dependent DNA polymerase activities by disrupting the enzyme's catalytic site. Viramune does not compete with template or nucleoside triphosphates, or inhibit the cellular DNA polymerases tested so far [21].

We first determined that the combination of Truvada and Viramune is effective against MLV. Using flow cytometry, we titrated the drug concentration for its ability to inhibit expression of green fluorescence protein encoded by MLV provirus upon infection; the EC_50 _was well below 100 nM (Figure 1A). When fed to Trex1-deficient mice at a dose comparable to that given to patients with HIV, the drugs substantially reduced mortality (Figure 1B). On sections of the heart from 9-month old treated mice, there were some mild patchy inflammatory infiltrates with little myocyte injury; but the difference to the marked inflammatory infiltrates with myocyte necrosis and dropout in 7-month old non-treated mice (at 9 months all untreated mice were dead) was striking (Figure 1C).

Almost half of the human genome consists of retroelements, many of them active. There are several ways that retroelements might trigger an autoimmune response, including (i) sensing of retroelement RNA and cDNA, (ii) generation of mimetopes through error-prone reverse transcription of mRNA encoding retroelement proteins, and (iii) insertional mutagenesis. We showed here that a hereditary autoimmune inflammation in the mouse that is likely caused by accumulation of retroelement cDNA can be treated with reverse transcriptase inhibitors. Other autoimmune diseases might be amenable to different interventions of retroelement activities.

## Abbreviations

AZT: zidovudine; AGS: Aicardi-Goutières syndrome; IFN: interferon; MLV: murine leukemia virus; SLE: systemic lupus erythematosus.

## Competing interests

The authors declare that they have no competing interests.

## Authors' contributions

GBE, DE, and MW planned the study; GBE carried out the experiments; and MW wrote the manuscript. All authors read and approved the final manuscript.
